# Application of the OliveID morphometric tool for the identification of archaeobotanical carbonized olive endocarps: evidence for morphological continuity with the modern Throumbolia cultivar

**DOI:** 10.3389/fpls.2026.1881563

**Published:** 2026-08-04

**Authors:** Konstantinos N. Blazakis, Ahsen Yüce, George Kostelenos, Panagiotis Kalaitzis

**Affiliations:** 1 Department of Horticultural Genetics and Biotechnology, Mediterranean Agronomic Institute of Chania (MAICh), Chania, Crete, Greece; 2 Kostelenos Olive Nurseries, Poros, Trizinia, Greece

**Keywords:** archaeobotany, carbonized olive stones, morphometrics, OliveID, Throumbolia

## Abstract

**Introduction:**

This study evaluates the applicability of digital morphometric analysis to images of archaeological carbonized olive endocarps as a proof-of-concept initial approach for archaeobotanical investigations. Conventional morphometric analyses of olive endocarps largely rely on manual measurements, limiting reproducibility and quantitative comparison.

**Methods:**

Ten archaeological endocarps were selected from previously published archaeological assemblages based on the integrity of their outlines, apex–base morphology and overall preservation quality. Quantitative descriptors describing endocarp size, symmetry, curvature and contour geometry were extracted using the OliveID software and compared with a modern morphometric reference database comprising Greek and international olive cultivars.

**Results:**

Reliable contour extraction and quantitative descriptor computation were successfully achieved for all archaeological specimens despite carbonization. Preliminary comparison of representative morphometric descriptors showed that the archaeological specimens were positioned within the morphometric variation observed among the modern reference collection. Hierarchical clustering consistently associated the archaeological endocarps with the modern Throumbolia morphotype, while distinguishing them from elongated, globular and mucro-bearing cultivars.

**Discussion:**

These findings demonstrate the feasibility of applying digital image-based morphometric analysis to sufficiently preserved archaeological carbonized olive endocarps and indicate a similar morphometric affinity between the analyzed archaeological material and the modern Throumbolia cultivar. This proof-of-concept study highlights the potential of digital morphometric approaches for quantitative archaeobotanical investigations of archaeological olive remains, while emphasizing the need for larger archaeological datasets and standardized image acquisition to further validate the observed morphometric similarity.

## 1 Introduction

The olive (*Olea europaea L. subsp. europaea* var. *europaea*) has been an emblematic fruit tree crop for the Mediterranean agriculture considering also the archaeobotanical evidence which indicated cultivation in Neolithic and Early Bronze Age (Margaritis, 2013; Livarda et al., 2021; Palli et al., 2025). Carbonized olive endocarps are frequently found well-preserved in archaeological sites, as demonstrated in olive stone assemblages in areas such as Tria Platania in northern Greece (Margaritis and Jones, 2008), providing valuable material for reconstructing olive germplasm diversity and cultivation practices across the Mediterranean.

Archaeobotanical studies from the Aegean and Crete highlighted the long-term presence of olive remains within complex agroecosystems and their integration into early agricultural systems (Livarda and Kotzamani, 2013; Valamoti et al., 2017; Henkel and Margaritis, 2024). These olive assemblages reflect both ecological variability and early human selection, resulting in diverse olive morphotypes shaped by environmental and cultural factors (Riley, 2004; Valamoti et al., 2018). Recent archaeobotanical investigations have further demonstrated the value of integrated archaeological datasets for reconstructing the emergence, management and spread of olive cultivation throughout the Aegean and the eastern Mediterranean. These studies provide an increasingly robust archaeological framework within which quantitative morphometric approaches can contribute to the investigation of olive domestication, cultivar diversification and long-term cultivation history (Ntinou and Valamoti, 2025; Terral et al., 2021).

The olive endocarp represents one of the most durable archaeobotanical structures owing to its highly lignified tissues and its resistance to mechanical degradation and moderate taphonomic alteration, allowing its frequent preservation in archaeological contexts (Margaritis and Jones, 2008; Märkle and Rösch, 2008; Ruas and Bouby, 2010). Its geometric features, such as size, symmetry, and curvature, contain taxonomically informative attributes that allow differentiation between wild and cultivated forms (Terral et al., 2004; Beyaz et al., 2017; Rallo et al., 2018). More broadly, morphometric analysis has become an established quantitative approach in archaeobotanical research, improving taxonomic resolution and enabling the investigation of domestication, crop evolution and varietal diversification across a wide range of archaeological plant remains (Portillo et al., 2020). Over the last two decades, morphometric approaches have been successfully applied to diverse archaeological plant remains, including cereal grains, grape seeds and olive endocarps, demonstrating that carbonized remains frequently preserve sufficient geometric information for quantitative analyses of taxonomy, domestication, diversification and crop evolution (Jeanty et al., 2024; Milanesi et al., 2014; Bonhomme et al., 2017; Terral et al., 2021; Roushannafas et al., 2023). However, traditional morphometric approaches rely on manual measurements, limiting reproducibility and comparability (Barranco et al., 2000; Trujillo et al., 2014).

Recent advances in digital image-based morphometrics, particularly through tools such as OliveID, enabled the extraction of quantitative descriptors from 2D images improving accuracy and reproducibility in cultivar discrimination and identification (Blazakis et al., 2017; Blazakis and Kalaitzis, 2023; Blazakis et al., 2024; Landa et al., 2021). In addition, large-scale morphometric reference datasets of olive pits have also been developed to facilitate cultivar discrimination and archaeobotanical applications (Ben-Dor et al., 2026). These approaches, combined with statistical and machine-learning methods, have been successfully applied to modern germplasm collections for cultivar classification (Gargiulo et al., 2025).

Three-dimensional (3D) imaging technologies have further expanded the possibilities for the quantitative characterization of olive fruits and endocarps. Three-dimensional morphometric approaches enable the extraction of volumetric and surface-related traits that cannot be fully captured using two-dimensional images improving cultivar discrimination and the quantitative characterization of fruit and stone morphology (Manolikaki et al., 2022; Sánchez-Beeckman et al., 2024; Gargiulo et al., 2025). Consequently, 3D image analysis represents a valuable complementary approach to conventional 2D morphometrics for olive phenotyping and cultivar characterization. Nevertheless, archaeological material is frequently available only as published photographs or legacy image collections, making standardized 3D acquisition difficult. In this case, robust two-dimensional morphometric approaches remain valuable for quantitative analysis of archaeological olive endocarps and for comparisons with modern reference collections. Future studies integrating standardized 3D imaging of original archaeological specimens with the present 2D morphometric framework may provide a more comprehensive assessment of morphological relationships and long-term phenotypic continuity between ancient and modern olive cultivars.

Although digital morphometric approaches have become increasingly established in archaeobotanical research and have been applied to a wide range of carbonized plant remains, few studies have evaluated the applicability of dedicated olive morphometric platforms to archaeological carbonized endocarps, particularly when analyses rely exclusively on previously published photographic material. Carbonization alters physical properties such as size and texture but may preserve relative geometric proportions under certain conditions (Märkle and Rösch, 2008; Braadbaart, 2008; Zhang et al., 2023). Recent studies emphasized that the application of morphometric approaches to charred olive material still requires further validation (Ben-Dor et al., 2026). Whether these features can be reliably captured and analyzed by using digital tools remains to be seen (Evin et al., 2020; Portillo et al., 2020; Tsirtsi et al., 2024). Preliminary results clearly demonstrated the applicability of digital morphometric analysis to archaeological carbonized olive endocarps (Blazakis et al., 2026).

Morphometric approaches have been extensively applied to carbonized archaeological cereal grains, demonstrating that charring frequently preserves sufficient geometric information for reliable taxonomic discrimination, species identification and the investigation of crop domestication and evolution. A wide range of quantitative methods including geometric morphometrics, elliptic Fourier analysis and multivariate image analysis were effective analyzing carbonized cereal remains despite the dimensional changes induced by charring (Jeanty et al., 2024; Milanesi et al., 2014; Bonhomme et al., 2017; Roushannafas et al., 2023). In other crops such as grapevine, the morphological traits remained stable over long periods of time indicating that morphometrics can be used for domestication and lineage continuity (Noraz et al., 2026; Bonhomme et al., 2021). It was shown that the phenotypic traits in vegetatively propagated olive cultivars are conserved (Trujillo et al., 2014; Besnard et al., 2018).

The aim of this report was the use of two-dimensional digital morphometric analysis on archaeological carbonized olive endocarps in order to investigate whether specimens retain sufficient morphological information for quantitative comparison with a modern olive cultivar reference database. The OliveID image analysis platform assessed the morphometric similarity of archaeological carbonized endocarps to a comprehensive collection of modern olive cultivars. This study might be considered a proof-of-concept approach demonstrating the feasibility of applying digital image-based morphometrics to archaeological olive remains and discusses its potential contribution to archaeobotanical research and studies of olive domestication history.

## 2 Materials and methods

### 2.1 Archaeological material

Carbonized olive stones were obtained from published archaeobotanical material. Eight specimens were digitally extracted (cropped) from a published photograph of the Tria Platania assemblage in northern Greece (Margaritis and Jones, 2008), which includes abundant charred olive remains recovered through flotation from Hellenistic contexts (4th–2nd century BC). Two additional specimens were obtained using the same procedure from published photographic material originating from Early/Late Minoan archaeological contexts in Crete (Hatzi-Vallianou, 2003). The original publication by Hatzi-Vallianou (2003) does not specify the exact archaeological locality of the illustrated carbonized endocarps used in the present study.

**Figure 1:**
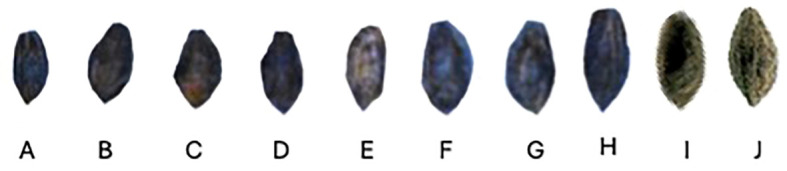
Selected olive stones which were used for the morphometric analysis. The first eight specimens **(A–H)** were discovered in Three Platania and the other two **(I, J)** in the region of Crete.

All specimens were selected based on the preservation quality of their outlines and the clear visibility of apex–base morphology, allowing reliable contour extraction and subsequent morphometric analysis. Only endocarps exhibiting continuous outlines, clearly identifiable apex and base regions, and no obvious evidence of fragmentation, severe deformation, or image artifacts that could compromise contour extraction were included in the analysis. The original published archaeological photographs, indicating the endocarps selected for morphometric analysis, are provided as **Supplementary Figures 1**, **2**. A total of ten well-preserved endocarps were included in the analytical dataset (**Figure 1**). Images of archaeological specimens were derived from previously published material (Margaritis and Jones, 2008; Hatzi-Vallianou, 2003), and no physical specimens were handled in this study.

The archaeological endocarps were quantitatively compared with the modern olive cultivar reference collection of the OliveID platform, comprising standardized images of authenticated Greek and international olive cultivars. Images in the reference collection were acquired under standardized imaging conditions according to the IOC/UPOV protocol and analyzed using the same image-processing workflow (Blazakis et al., 2017; Blazakis and Kalaitzis, 2023; Blazakis et al., 2024). The objective of the specimen selection was not to obtain a representative archaeological sample, but to identify published archaeological endocarps suitable for testing the feasibility of automated contour extraction and digital morphometric analysis.

### 2.2 Endocarps of olive cultivars

Endocarp samples of Greek and international olive cultivars were collected from olive trees grown at the Kostelenos nursery (Poros, Trizinia, Greece) during two consecutive growing seasons (2016–2017). The modern reference collection was developed as part of the OliveID digital morphometric platform (Blazakis et al., 2017; Blazakis and Kalaitzis, 2023; Blazakis et al., 2024). For each cultivar, a minimum of 50 mature endocarps collected from trees under normal fruit load were analyzed to establish the cultivar morphometric reference profile based on the distribution of the OliveID quantitative descriptors. Normal fruit load refers to trees exhibiting a typical crop load for the respective cultivar under standard orchard management conditions, thereby minimizing the influence of abnormal fruiting intensity on endocarp morphology. The complete list of cultivars included in the OliveID reference database has been described in detail in Blazakis et al. (2017) and Blazakis et al. (2024).

Endocarps were extracted from fruits harvested at the breaker stage from five trees per cultivar. Fruits were sampled from the mid-shoot portion of the current year’s growth around the canopy at approximately 1.5 m above ground level. Only trees exhibiting normal fruit load were included. After pulp removal using coarse fabric, the endocarps were disinfected in 10% NaOCl for 5 min and stored under dry conditions until imaging. For each cultivar, 50 fruits, 50 endocarps and 25 leaves were analyzed according to the standardized OliveID workflow.

### 2.3 Image acquisition and morphological analysis

Each endocarp of the modern olive cultivars was photographed using a copy stand and uniform diffuse light approximating daylight (5400 K). Images were captured in two orientations following the IOC/UPOV guidelines: Position A, displaying maximum asymmetry with the carpel suture facing the observer, and Position B, rotated 90°from Position A. All images were stored in high-resolution PNG (300dpi) format with a scale bar for calibration.

The archaeological endocarp images were obtained from previously published archaeobotanical studies and, therefore, were not acquired according to the standardized IOC/UPOV imaging protocol. Consequently, the orientation of the archaeological specimens could not be controlled. Comparisons were performed against the modern reference database, which included images acquired in both IOC/UPOV standard orientations (Position A and Position B). Only archaeological endocarps exhibiting clearly distinguishable outlines, apex and base regions, and sufficient image quality to allow reliable contour extraction were selected for morphometric analysis.

Digital contour extraction and computation of geometric descriptors were performed using the OliveID software. Similar image-processing workflows and morphometric descriptor extraction approaches have recently been applied for the development of large-scale olive pit reference datasets intended for cultivar discrimination and archaeobotanical applications (Ben-Dor et al., 2026). Although OliveID is capable of extracting morphometric information from olive leaves, fruits, and endocarps, only the endocarp morphometric dataset was used in the present study. Accordingly, all statistical analyses were performed exclusively using quantitative descriptors extracted from endocarp images. The complete image-processing workflow and computation of the morphometric descriptors have been described in detail by Blazakis et al. (2017). Briefly, each image was segmented to isolate the endocarp from the background, after which the endocarp contour was automatically extracted. Based on the extracted contour, the software automatically computed quantitative morphometric descriptors describing endocarp size, shape, symmetry, curvature, and contour geometry. A schematic overview of the OliveID image analysis workflow is provided in **Supplementary Figure 3**, while a detailed description of the nineteen quantitative morphometric descriptors used in the present study is provided in **Supplementary Table 1**. The extracted variables included projected area, perimeter, height, maximum transversal diameter, minimum contour-to-transversal diameter distance (minCTD), vertical symmetry, transversal symmetry, shape index, major and minor ellipse axes, apex and base area, apex and base length, apex and base curvature, endocarp circularity, ellipse circularity, and circularity ratio. Because the OliveID workflow is based on two-dimensional images, the extracted descriptors characterize the planar geometry of the endocarp and do not capture dorsal–ventral asymmetry or volumetric characteristics, which require three-dimensional imaging approaches (Terral et al., 2004; Bourgeon et al., 2018).All variables were standardized using z-score normalization prior to multivariate statistical analyses (Kaufman and Rousseeuw (2009).

For the preliminary quantitative comparison between archaeological and modern material, six representative morphometric descriptors were selected: projected area, vertical symmetry, transversal symmetry, shape index, apex curve area and endocarp circularity. These descriptors were chosen because they represent complementary aspects of endocarp size, symmetry, shape, apex morphology and overall contour geometry, and have previously been shown to be among the most informative descriptors for olive cultivar discrimination using the OliveID platform (Blazakis et al., 2024).

### 2.4 Statistical analysis

A pairwise Euclidean distance matrix was calculated across all modern and archaeological samples. Cluster analysis was performed using the UPGMA (Unweighted Pair Group Method with Arithmetic Mean) algorithm implemented in MATLAB. Bootstrap resampling with 500 replicates provided branch support values. Six key descriptors—area, vertical symmetry, transversal symmetry, shape index, apex curve area, and average circularity—were used for comparative barplots of mean values between carbonized specimens and the modern Throumbolia, based on their high discriminatory power reported in previous classification studies (Blazakis et al., 2024).

## 3 Results

### 3.1 Preliminary comparison of morphometric descriptors

**Figure 2:**
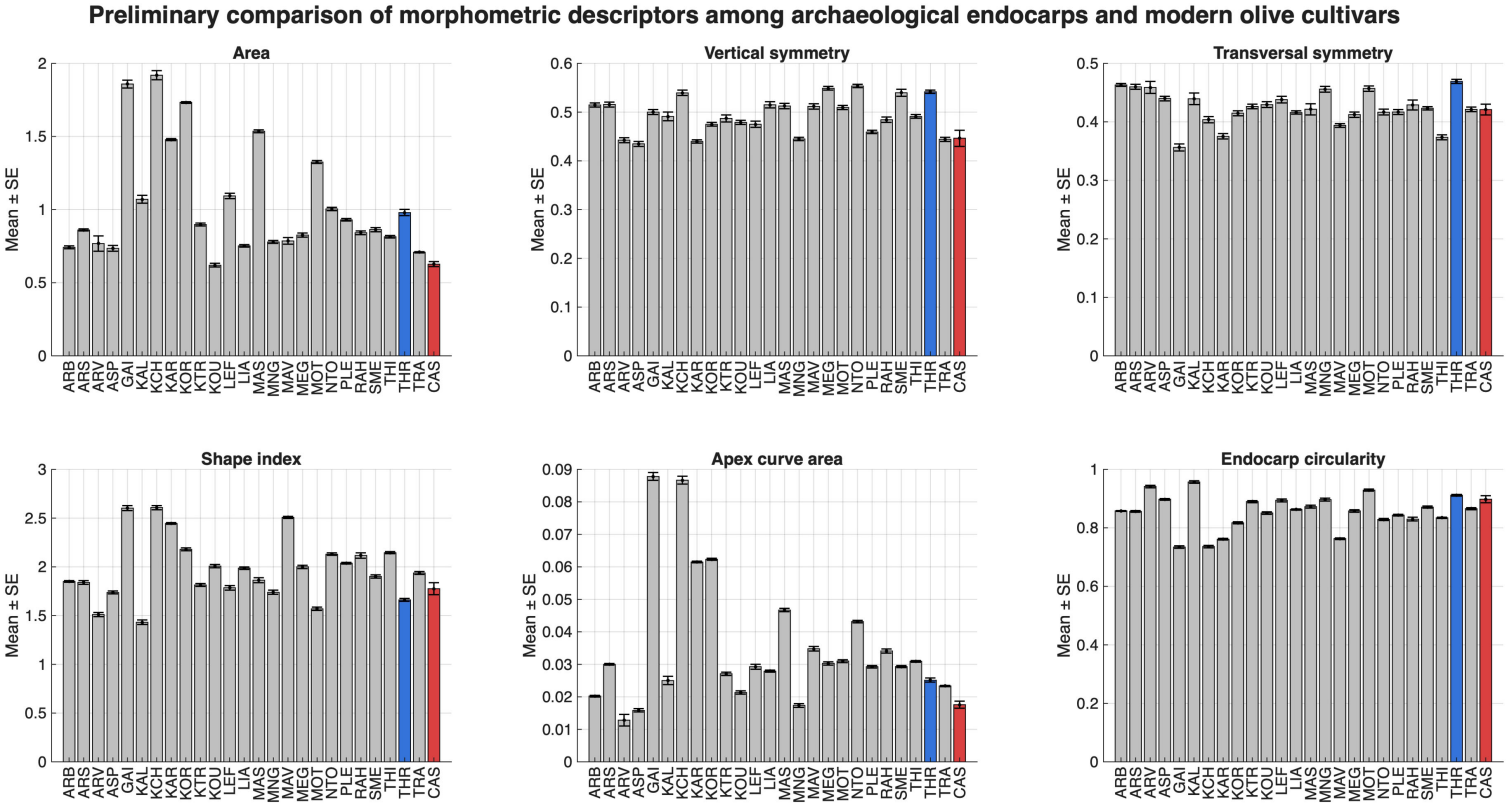
Preliminary comparison of six representative morphometric descriptors between archaeological carbonized olive endocarps and modern olive cultivars. Mean values ± standard error are presented for all modern cultivars included in the reference database. A three-letter acronym was used to name each cultivar in the dendrogram: ARB, Arbequina; ARS, Arbosana; ARV, Arvanitolia; ASP, Asprolia Alexandroupolis; GAI, Gaidourelia; KAL, Kalamon; KCH, Karidolia Chalkidikis; KAR, Karidolia Trizinias; KOR, Koroneiki; KOU, Koutsourelia; KTR, Kothreiki; LEF, Lefkolia Serron; LIA, Lianomanako Tyrou; MAS, Mastoidis; MNG, Mastoidis N, K Gigas; MAV, Mavrolia Serron; MEG, Megaron; MOT, Mothonia Egialias; NTO, Ntopia Atsiholou; PLE, Plexidenia; RAH, Rahati; SME, Smertolia; THI, Thiaki; THR, Throumbolia; TRA, Tragolia. The Throumbolia cultivar (THR) is highlighted in blue, while the archaeological carbonized endocarps (CAS) are highlighted in red. The figure provides an initial descriptive comparison prior to the multivariate morphometric analyses.

The images of the carbonized stones were comprised of archaeological assemblages derived from published archaeobotanical material of specimens from Tria Platania in northern Greece (Margaritis and Jones, 2008) as well as additional material from the region of Crete (Hatzi-Vallianou, 2003). Initially six representative morphometric descriptors were determined and compared between the archaeological carbonized endocarps and the complete modern olive cultivar reference collection (**Figure 2**). The archaeological specimens were positioned within the range of variation observed among the modern cultivars for all descriptors examined. Regarding endocarp size (Area), the archaeological specimens exhibited intermediate values that fell within the morphometric variation observed across the modern reference collection. Vertical and transversal symmetry values were comparable to those observed in several modern cultivars, with particularly close agreement to the Throumbolia cultivar for transversal symmetry.

The archaeological endocarps also displayed intermediate shape index values, indicating a general endocarp elongation similar to that observed in numerous cultivated olive varieties. Apex curve area was relatively small and comparable to the values recorded for Throumbolia and several additional cultivars, indicating that sufficient apex geometry was preserved to allow reliable morphometric characterization. The endocarp circularity of archaeological specimens showed extensive similarity to Throumbolia cultivar, although partial overlap with additional cultivars was also observed. These results indicate that individual morphometric descriptors are not sufficient for unequivocal cultivar discrimination. However, taken together, they demonstrate that the archaeological specimens are consistently positioned within the range of morphometric variation observed among the modern olive cultivars. The quantitative measurements of the archaeological specimens are presented in **Supplementary Table 2**, whereas the corresponding mean values and standard errors for the modern cultivars are provided in **Supplementary Table 3**. The archaeological specimens exhibited relatively limited variation across the examined descriptors, with all ten endocarps being positioned within the morphometric variation observed among the modern reference collection. This preliminary quantitative characterization provided additional support for the subsequent hierarchical clustering analysis based on the complete set of morphometric descriptors.

### 3.2 Preservation status of archaeological stones morphotypes

**Figure 3:**
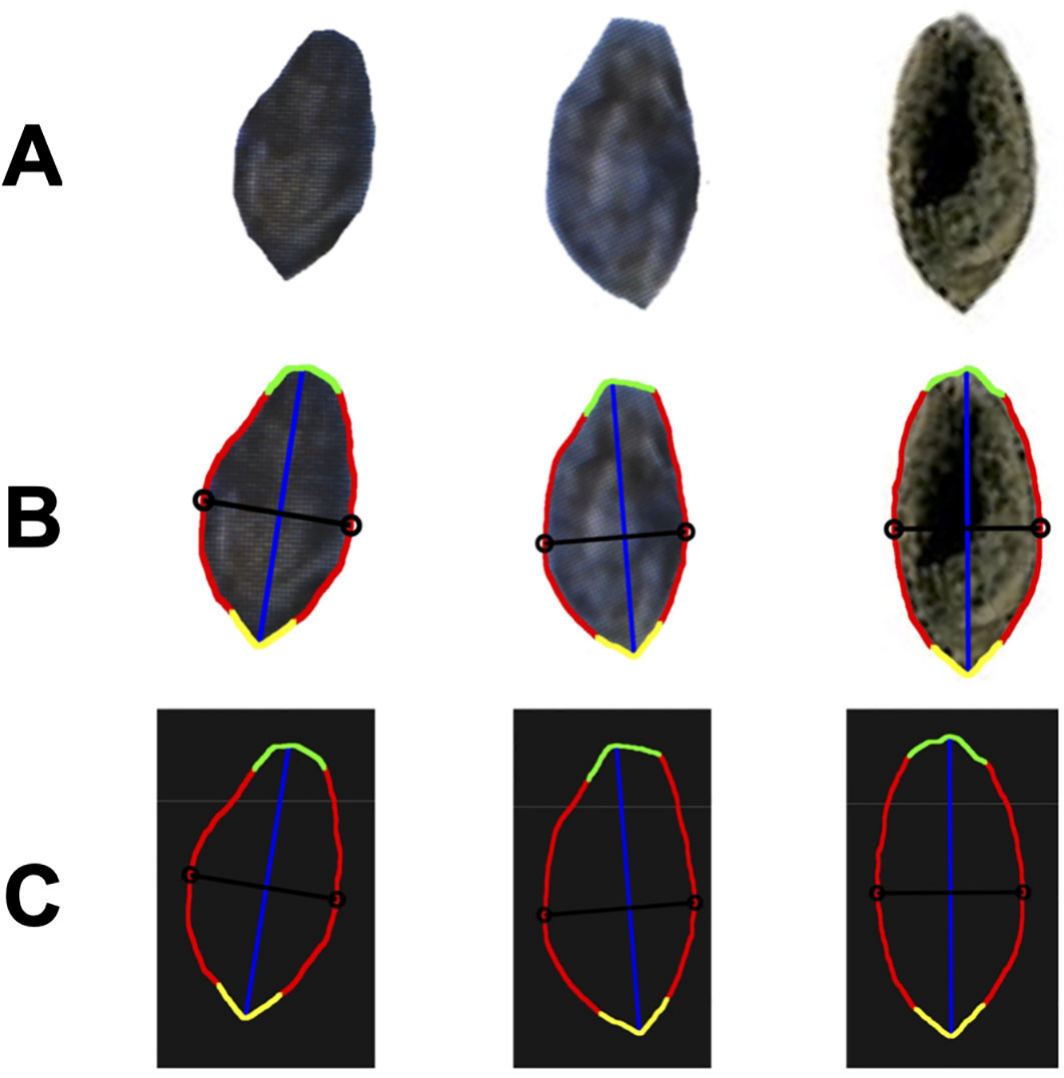
OliveID processing of carbonized archaeological olive endocarps selected from **Figure 1** (endocarps B, F, and I, respectively). Original images of carbonized olive stones **(A)**, geometric feature extraction overlaid on the images **(B)**, and corresponding contour representations **(C)**. The extracted contour (red), longitudinal axis (blue), and maximum transversal diameter (black) define the principal geometry, with apex and base indicated in yellow and green. The continuous contours and consistent geometric features demonstrate that OliveID can reliably extract morphometric information from carbonized endocarps.

The archaeological assemblages of olive specimens exhibited a wide range of preservation states, including surface darkening, minor abrasion, adhering sediments, and variable carbonization-induced shrinkage, defined here as the reduction in endocarp dimensions resulting from the carbonization process (**Figure 3**). Despite this heterogeneity, ten archaeological endocarps fulfilled the predefined selection criteria, exhibiting continuous outlines, clearly distinguishable apex and base regions, no obvious fragmentation or severe deformation, and sufficient image quality for reliable contour extraction. These specimens were therefore selected for all subsequent morphometric analyses.

These specimens exhibited continuous, well-defined outlines without edge fragmentation or surface detachment. The apex and basal regions remained clearly distinguishable, and no evidence of torsion, lateral compression, or planar deformation was observed—conditions that could otherwise compromise symmetry- and curvature-based descriptors.

The preserved stones maintained key aspects of their geometric architecture, including gradual apical tapering, a rounded base, smooth lateral margins, and an overall elongated–elliptic shape (**Figure 2**). Their contours remained sharply defined, without warping or angular collapse that could interfere with boundary detection. The OliveID algorithm automatically extracted continuous contours and generated stable curvature profiles for all selected stones, enabling the computation of all six high-discriminant descriptors without manual intervention (**Figure 3**).

No algorithmic artifacts—such as contour discontinuities, segmentation noise, or axis misalignment—were observed during processing. This indicates that the geometric integrity of the stones is sufficiently preserved to support automated morphometric analysis. Despite alterations in surface texture caused by carbonization, the archaeological endocarps retained continuous outlines and clearly identifiable geometric landmarks (apex, base and lateral margins), allowing reliable contour extraction and quantitative morphometric analysis. This observation is further supported by the quantitative similarity of the extracted morphometric descriptors to those of the modern reference collection (**Figure 2**; **Supplementary Tables 2**, **3**).

The consistent identification of key morphological landmarks, including apex position, basal morphology, and lateral curvature, further confirms that the underlying morphological signal is retained. As a result, the selected specimens can be analyzed using the same computational framework applied to regular olive stone reference material. The carbonized olive endocarps, when sufficiently preserved, can be reliably processed using the OliveID pipeline, establishing a robust basis for subsequent comparative morphometric analyses (**Figure 1**).

### 3.3 Morphological structure of olive cultivars stones

**Figure 4:**
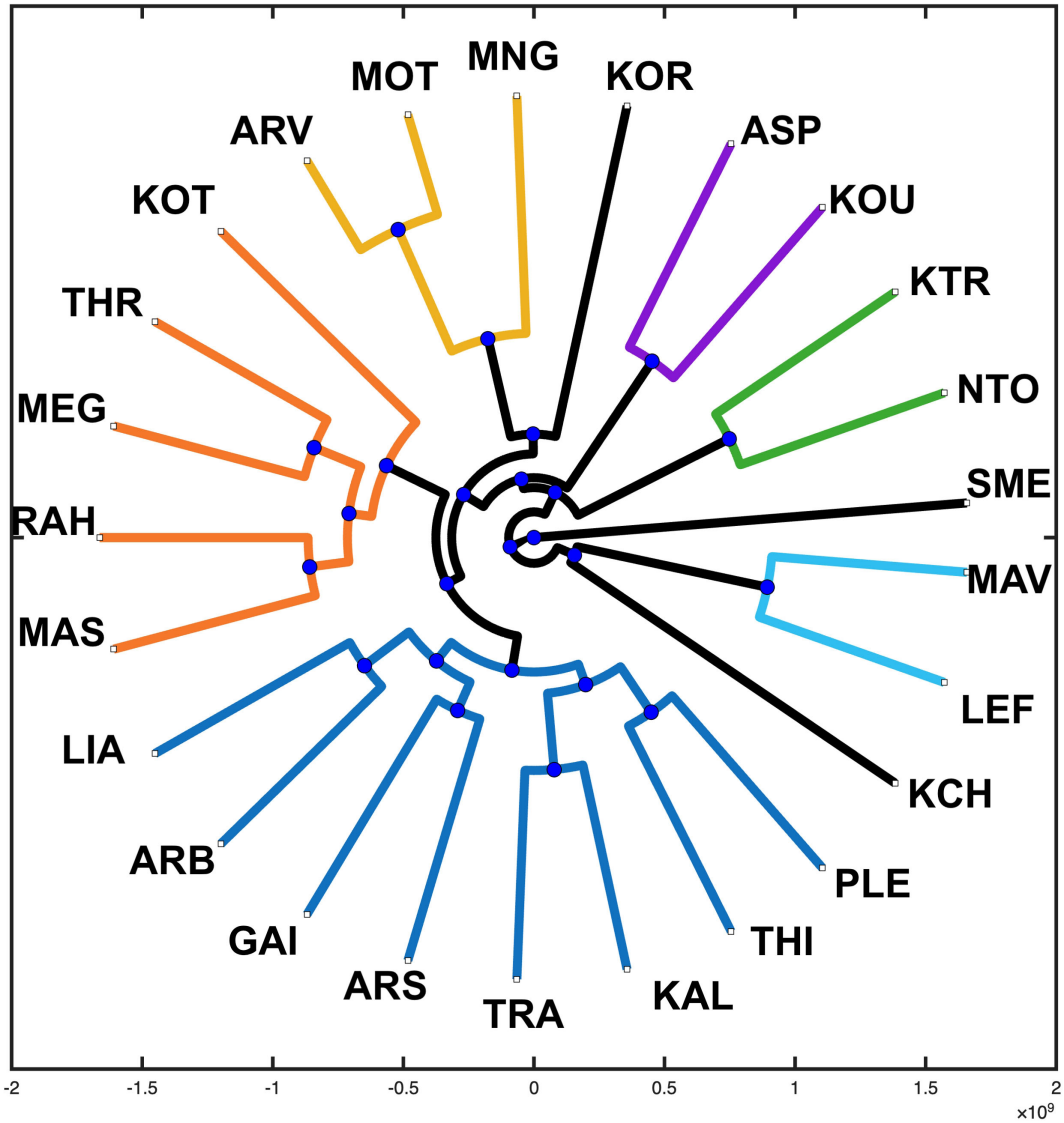
Radial hierarchical clustering of olive cultivars based on endocarp morphometric descriptors. The dendrogram shows the relationships among cultivars based on Euclidean distances calculated from OliveID descriptors using the UPGMA algorithm. A three-letter acronym was used to name each cultivar in the dendrogram: ARB, Arbequina; ARS, Arbosana; ARV, Arvanitolia; ASP, Asprolia Alexandroupolis; GAI, Gaidourelia; KAL, Kalamon; KCH, Karidolia Chalkidikis; KAR, Karidolia Trizinias; KOR, Koroneiki; KOU, Koutsourelia; KTR, Kothreiki; LEF, Lefkolia Serron; LIA, Lianomanako Tyrou; MAS, Mastoidis; MNG, Mastoidis N, K Gigas; MAV, Mavrolia Serron; MEG, Megaron; MOT, Mothonia Egialias; NTO, Ntopia Atsiholou; PLE, Plexidenia; RAH, Rahati; SME, Smertolia; THI, Thiaki; THR, Throumbolia; TRA, Tragolia. Blue nodes represent internal clustering points (merging events) in the hierarchical analysis, reflecting the similarity levels among samples.

The broad morphometric diversity of the current Greek olive germplasm might be represented in the hierarchical clustering of 27 Greek cultivars suggesting distinct morphological groups (**Figure 4**). Morphometric analysis was performed on olive endocarps using the OliveID platform (Blazakis et al., 2017), which extracted quantitative descriptors from the two-dimensional outline of each stone. These descriptors included size-related traits such as projected area and perimeter, shape descriptors such as length-to-width ratio expressed as shape index and elliptic variance, symmetry parameters such as vertical and transversal symmetry, curvature-related metrics describing the apex, base, and overall curvature distribution, as well as contour descriptors such as circularity and convexity. Large-fruited cultivars such as Karidolia-Chalkidikis, Mavrolia-Serron, and Lefkolia-Serron form a coherent cluster, reflecting their increased endocarp size and broader geometry. Cultivars such as Koutsourelia and Asprolia-Alexandroupolis are clustered in a separate group, corresponding to differences in apex morphology and overall shape configuration. Throumbolia grouped together with cultivars including Megaron, Rahati, and Mastoidis, forming a distinct cluster characterized by similar shape-index values and comparable elongation patterns. Strongly elongated cultivars such as Koroneiki are positioned separately, consistent with their higher shape-index values and slenderer outline. A broader group included cultivars such as Arbequina, Arbosana, Kalamon, and Gaidourelia which formed an additional cluster, reflecting intermediate morphometric characteristics between elongated and large-fruited morphotypes. The overall clustering of olive cultivars demonstrated that endocarp morphology is highly structured and cultivar-specific, with minimal overlap among groups which exhibit divergent geometric features (**Figure 3**).

### 3.4 Morphometric profiles of the archaeological stone specimens

**Figure 5:**
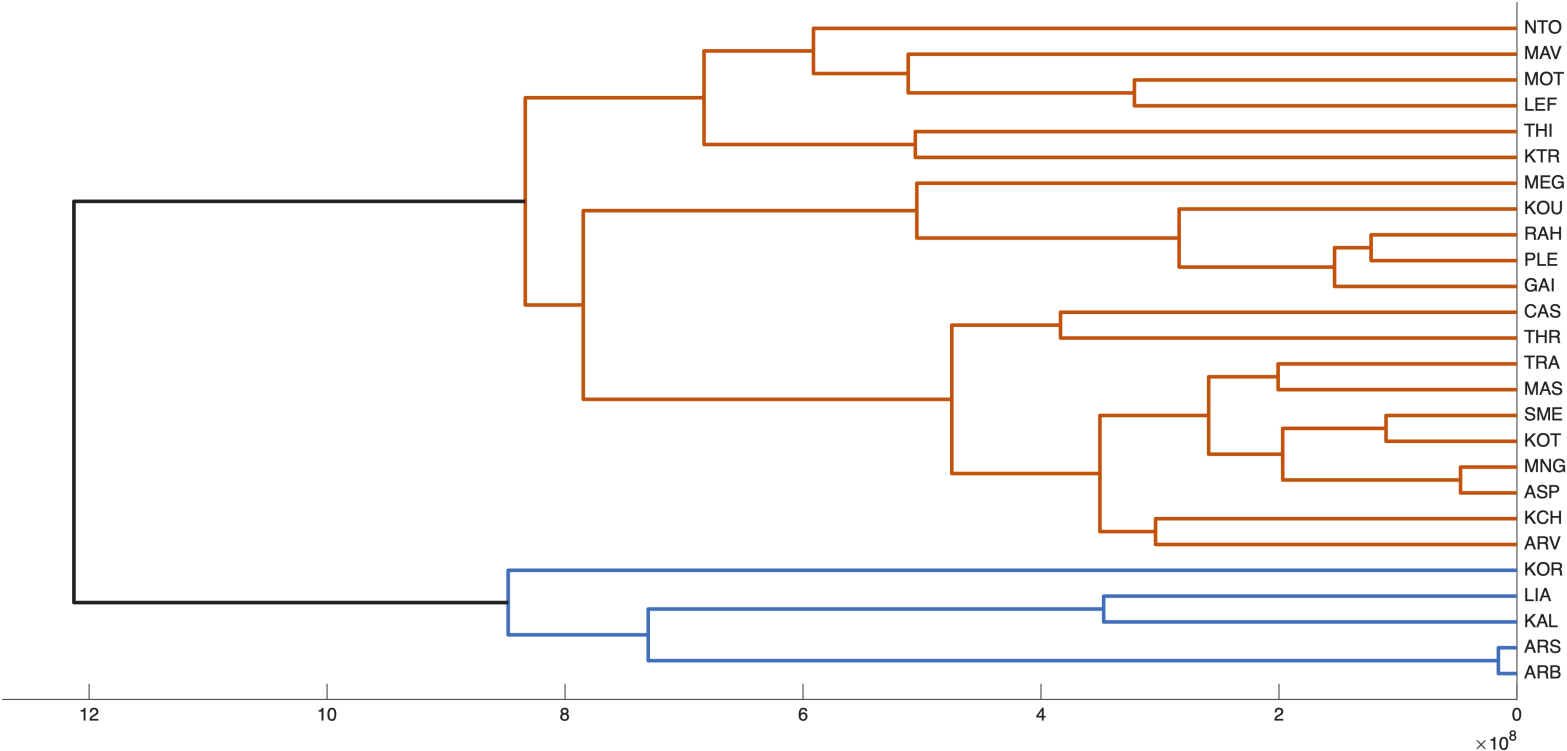
Hierarchical clustering based on comparative morphological descriptors of archaeological stones within the diversity context of 27 Greek olive cultivars. The average values of all ten archaeological specimens were used for the dendrogram construction. A three-letter acronym was used to name each cultivar in the dendrogram: ARB, Arbequina; ARS, Arbosana; ARV, Arvanitolia; ASP, Asprolia Alexandroupolis; GAI, Gaidourelia; KAL, Kalamon; KCH, Karidolia Chalkidikis; KAR, Karidolia Trizinias; KOR, Koroneiki; KOU, Koutsourelia; KTR, Kothreiki; LEF, Lefkolia Serron; LIA, Lianomanako Tyrou; MAS, Mastoidis; MNG, Mastoidis N, K Gigas; MAV, Mavrolia Serron; MEG, Megaron; MOT, Mothonia Egialias; NTO, Ntopia Atsiholou; PLE, Plexidenia; RAH, Rahati; SME, Smertolia; THI, Thiaki; THR, Throumbolia; TRA, Tragolia; CAS, Carbonized Stones. Blue nodes represent internal clustering points (merging events) in the hierarchical analysis, reflecting the similarity levels among samples.

The archaeological specimens were analyzed using multivariate hierarchical clustering based on the complete morphometric profile (**Figure 5**) after the preliminary descriptor-level comparison (**Figure 2**). Although several cultivars exhibited overlap with the archaeological specimens for individual descriptors (**Figure 2**), simultaneous analysis of all six descriptors clearly associated the archaeological material with the modern Throumbolia morphotype (**Figure 5**). The comparison and clustering among the archaeological endocarps and olive cultivars was based on six OliveID descriptors such as area, vertical symmetry, transversal symmetry, shape index, apex-curve area, and circularity (**Figure 5**). These morphological descriptors were selected because represented independent and biologically meaningful features of endocarp morphology, including size, elongation, bilateral symmetry, apex development, and contour regularity. Moreover, these descriptors have been identified as among the most informative for olive cultivar discrimination in recent morphometric and machine-learning analyses (Blazakis et al., 2024). The average morphological descriptors values of the archaeological specimens cluster together with the modern Throumbolia cultivar in the dendrogram due to similar values in shape index, symmetry parameters, and circularity, indicating comparable overall geometry (**Figure 5**). In the same cluster, there was another subgroup comprised of Tragolia and Mastoidis as well as Smertolia, Kothreiki, Mastoidis N-K-Gigas, Asprolia-Alexandroupolis, Karidolia-Chalkidikis, and Arvanitolia which shared overlapping values of symmetry and curvature-related descriptors, particularly in apex curvature and circularity. However, Koroneiki was grouped together with Lianomanako, Kalamon, Arbequina and Arbosana in another major cluster distant from all the other cultivars due to higher shape index values and more elongated, slender endocarp morphology (**Figure 5**). Within the Koroneiki cluster, the Lianomanako Tyrou and Kalamon as well as Arbosana and Arbequina create two subgroups reflecting their relatively smaller size, due to lower area values, and higher circularity which corresponded to more compact and less elongated endocarp shapes (**Figure 5**).

A distinct subcluster was comprised of the olive cultivars of Ntopia-Atsiholou, Mavrolia-Serron, Mothonia-Egialias, and Lefkolia-Serron, due to relatively higher shape index values combined with moderate symmetry and intermediate circularity, indicating more elongated and less compact endocarp forms (**Figure 5**). In the same subcluster, the olive cultivars of Thiaki and Karidolia Trizinias were grouped together consistent with similar elongation patterns and comparable symmetry parameters (**Figure 5**). Another distinct subcluster was comprised of the olive cultivars of Megaron, Koutsourelia, Rahati, Plexidenia, and Gaidourelia, due to their intermediate morphometric characteristics, particularly moderate shape index values and relatively balanced vertical and transversal symmetry, indicating neither strongly elongated nor highly compact endocarp forms (**Figure 5**).

The dendrogram indicated that endocarp morphology was structured in distinct clusters reflecting differences in size, symmetry, elongation, and curvature-related traits (**Figure 5**).

### 3.5 Dendrogram comprising individual archaeological stones

**Figure 6:**
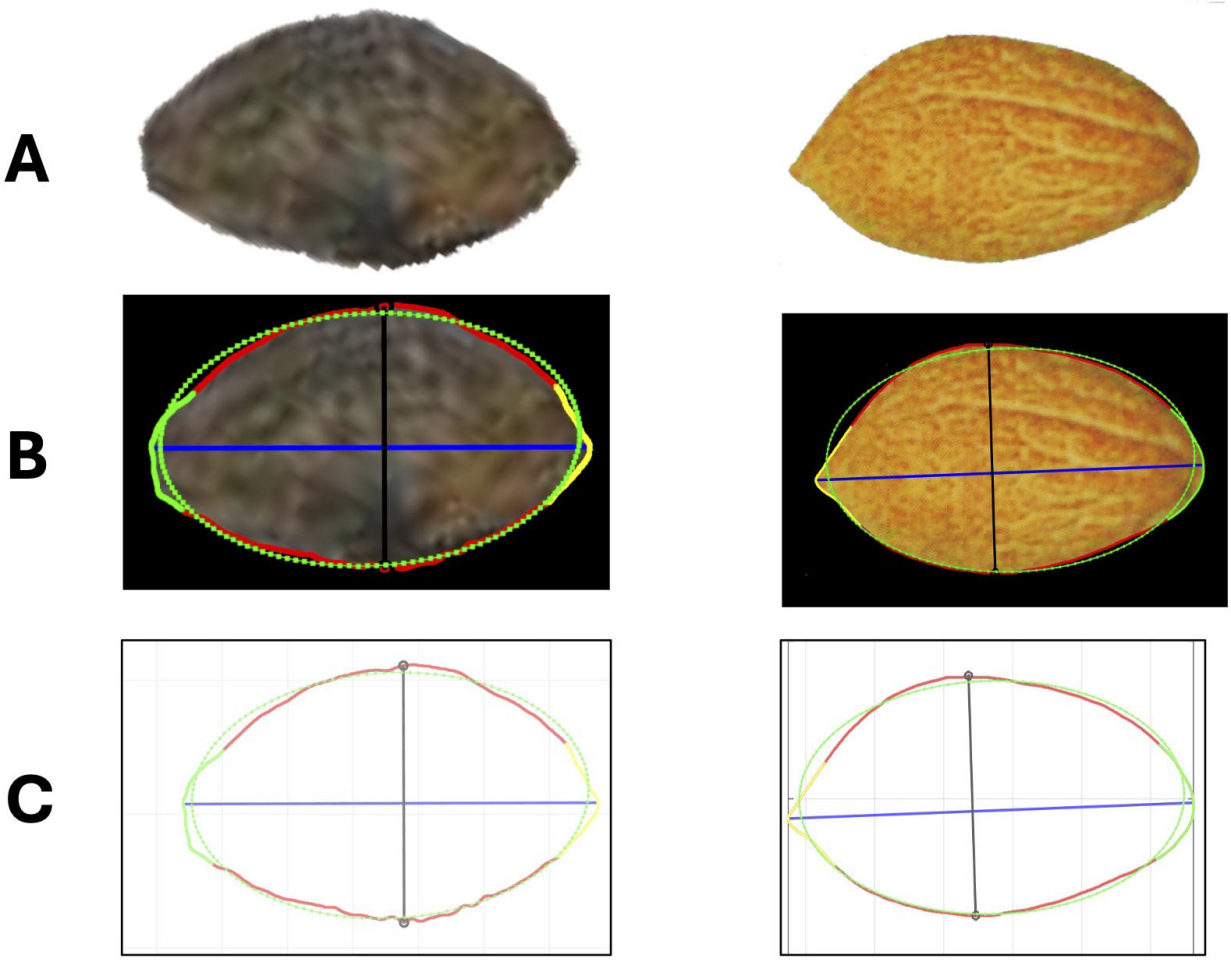
Radial hierarchical clustering of archaeological stones (CS1–CS10) and modern Greek cultivars based on morphometric descriptors. Archaeological, carbonized stones are denoted as CS1–CS10. A three-letter acronym was used to name each cultivar in the dendrogram: ARB, Arbequina; ARS, Arbosana; ARV, Arvanitolia; ASP, Asprolia Alexandroupolis; GAI, Gaidourelia; KAL, Kalamon; KCH, Karidolia Chalkidikis; KAR, Karidolia Trizinias; KOR, Koroneiki; KOU, Koutsourelia; KTR, Kothreiki; LEF, Lefkolia Serron; LIA, Lianomanako Tyrou; MAS, Mastoidis; MNG, Mastoidis N, K Gigas; MAV, Mavrolia Serron; MEG, Megaron; MOT, Mothonia Egialias; NTO, Ntopia Atsiholou; PLE, Plexidenia; RAH, Rahati; SME, Smertolia; THI, Thiaki; THR, Throumbolia; TRA, Tragolia; Blue nodes represent internal clustering points (merging events) in the hierarchical analysis, reflecting the similarity levels among samples.

The geometric descriptors which were defined solely from the image of each one of the ten carbonized stones were compared with those of the 27 Greek olive cultivars by using hierarchical clustering (**Figure 6**). All ten stones were clustered exclusively with those of Throumbolia cultivar creating a distinct group (**Figure 6**).

The dendrogram indicated that all ten specimens exhibited morphometric similarity to Throumbolia morphological features (**Figure 6**). Moreover, it indicated the lack of variability within the ten specimens (**Figure 6**). None of the archaeological specimens was clustered with any other Greek cultivar or sub-group corresponding to elongated, mucro-bearing, or large-fruited cultivars (**Figure 6**).

This result is in alignment with the preliminary quantitative comparison (**Figure 2**) demonstrating that the combined morphometric information, rather than any single descriptor, provides sufficient discriminatory power for the archaeological specimens. The observed morphometric similarity was supported by both the preliminary descriptor-level comparison and the individual hierarchical clustering analyses. While the average descriptor values indicated extensive similarity between the archaeological specimens and the modern Throumbolia cultivar, the hierarchical clustering further demonstrated that each archaeological endocarp was consistently similar to the Throumbolia morphotype based on its complete multivariate morphometric profile.

The characterization of variability within the stones assemblages showed that the ten archaeological endocarps clustered within an exceptionally homogeneous morphometric group. The archaeological endocarps exhibited similar morphometric profiles based on the six predefined morphometric descriptors described in the Materials and Methods. The absence of outliers within the carbonized stones in the dendrogram suggested a morphologically stable cluster and not a mixture of diverse olive germplasm stones.

The intra-olive stone assemblage variance is comparable to that observed among Throumbolia cultivar stones. Therefore, the carbonized specimens are mostly aligned with the Throumbolia stone morphotype. The archaeological stones represented a coherent morphotype within the modern Throumbolia morphological profile across all six diagnostic descriptors showing no similarity with other cultivars.

The combined results from morphological preservation assessment, descriptor-level comparisons, and individual-level hierarchical clustering reveal a highly consistent and robust pattern. The archaeological endocarps retain sufficient geometric integrity for high-resolution morphometric analysis and reproduce in detail the full morphometric signature of the modern Throumbolia cultivar.

### 3.6 Morphometric comparison to Throumbolia

**Figure 7:**
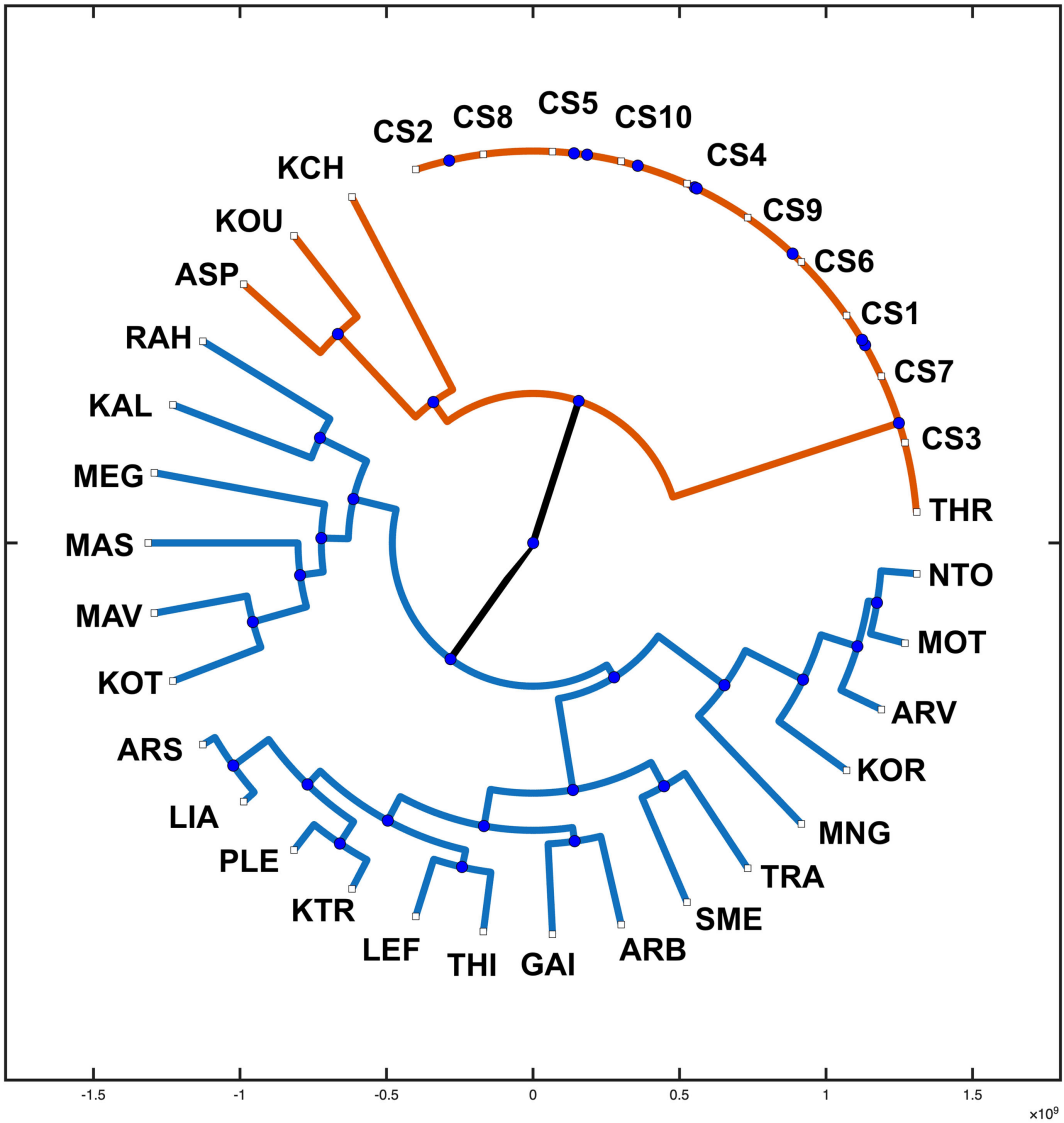
Morphometric comparison between an archaeological olive specimen and a modern Throumbolia stone. The archaeological specimen is shown alongside a modern Throumbolia reference **(A)**. The OliveID-derived contour (red), longitudinal axis (blue), and transversal axis (black) capture the principal geometry, with apex and base indicated in yellow and green **(B)**. The close agreement in shape, elongation, and curvature highlights the strong morphological similarity between archaeological and modern material **(C)**.

The archaeological specimens were directly compared to modern endocarps of the Throumbolia cultivar (**Figure 7**). A strong visual similarity was observed among the archaeological and Throumbolia stones sharing highly similar aspect ratios, apical tapering, and smooth lateral curvature (**Figure 6**). The overall geometry of the archaeological specimens consistently conforms to the elongated–elliptic morphotype characteristic of Throumbolia (**Figure 7**).

This similarity was further supported by the OliveID, according to which the extracted contours closely match the specimen outlines and preserve the same geometric proportions (**Figure 7**). The alignment of the longitudinal and transversal axes, together with the consistent identification of apex and basal regions, confirmed that the principal morphological dimensions of the archaeological stones closely corresponded to those of the modern Throumbolia reference material (**Figure 7**).

The archaeological stones did not exhibit the irregular protrusions, asymmetries, or lobed contours found in other Greek cultivars, suggesting that their fundamental morphotype was preserved despite carbonization. Their shape consistently matched the morphometric domain defined by modern Throumbolia.

## 4 Discussion

### 4.1 Reliability of digital morphometrics for archaeobotanical material

The structural integrity of the carbonized olive specimens used in this study was comparable to previous archaeobotanical assemblages in other Mediterranean archaeological contexts in which carbonized stones often retained sufficient morphological integrity for morphometric analysis (Margaritis and Jones, 2008). The images of the archaeological specimens showed sufficient fidelity in outline shape, apex morphology, bilateral symmetry, and overall contour geometry to support reliable morphometric comparison with modern cultivars (**Figure 1**).These descriptors have been identified as among the most informative for olive cultivar discrimination in recent morphometric and machine-learning analyses (Blazakis et al., 2024). In addition, the extracted contours showed continuous outlines without obvious discontinuities, severe distortions or contour irregularities that could compromise the computation of the morphometric descriptors (Evin et al., 2020; Portillo et al., 2020; Tsirtsi et al., 2024). This is further supported by the successful automatic extraction of the endocarp contours and the subsequent computation of all quantitative descriptors for the selected archaeological specimens (Blazakis et al., 2017). Although carbonization alters color, surface texture, and absolute dimensions, previous experimental and archaeological studies have shown that relative geometric proportions can remain stable under moderate charring conditions (Märkle and Rösch, 2008; Ruas and Bouby, 2010).

The archaeological specimens in this report (**Figure 1**), derived from published material originating from both northern Greece (Margaritis and Jones, 2008) and the region of Crete (Hatzi-Vallianou, 2003), preserved continuous outlines, well-defined apices, and undistorted lateral margins. This level of preservation allowed the OliveID workflow (Blazakis et al., 2017; Blazakis and Kalaitzis, 2023; Blazakis et al., 2024) to automatically extract stable endocarp contours and compute the corresponding quantitative morphometric descriptors without manual intervention. The successful automated processing of the specimens further indicated that shape-based descriptors remained informative even after carbonization, provided that taphonomic distortion was limited (Gargiulo et al., 2025; Tsirtsi et al., 2024). Similar applications of geometric morphometrics on archaeological olive assemblages have demonstrated the capacity of shape-based approaches to characterize ancient varietal diversity and cultivation practices (Pagnoux et al., 2026). Image-based morphometrics can serve as a reproducible and objective tool in archaeobotanical investigations, bridging traditional plant archaeology and quantitative plant phenotyping. These findings are consistent with the increasing adoption of geometric morphometrics in archaeobotanical research, where quantitative shape analysis has been successfully applied to cereal grains, grape seeds and olive endocarps to investigate taxonomy, domestication and crop diversification (Bonhomme et al., 2017; Portillo et al., 2020; Terral et al., 2021). Within this broader methodological framework, the present study extends the application of digital morphometrics to archaeological carbonized olive endocarps using previously published photographic material. The present findings extend recently developed olive pit morphometric reference frameworks (Ben-Dor et al., 2026) by demonstrating that sufficiently preserved carbonized archaeological endocarps can retain stable cultivar-level geometric information suitable for automated morphometric analysis.

Recent archaeobotanical research across the Mediterranean has increasingly emphasized the importance of combining quantitative morphometric methods with contextual archaeological evidence in order to reconstruct past agricultural practices and plant genetic resources exploitation strategies (Terral et al., 2004; Livarda et al., 2021; Henkel and Margaritis, 2024). In this context, extensive morphological analysis of archaeological carbonized stones might provide additional information regarding the origin of these specimens taking into consideration that just the use of specimens images might identify their cultivar origin. Previous results indicated that endocarp morphological features have the greatest capacity for cultivar discrimination since these traits are less affected by the variability of environmental conditions (Blazakis et al., 2024).

### 4.2 Morphological affinity and the issue of convergent forms

The archaeological assemblage from Crete is represented by only two endocarps. Although these specimens are morphometrically similar to the Throumbolia morphotype and to those endocarps identified in the northern Greek assemblage, this result might be considered preliminary. Additional archaeological material from Crete and other regions have to be used to characterize the spatial distribution and long-term continuity of this morphotype.

The preliminary descriptor-level comparison (**Figure 2**) showed that several individual morphometric variables of the archaeological specimens overlapped with those of many cultivars, indicating that no single descriptor alone was sufficient for cultivar identification. However, integration of all descriptors in the hierarchical clustering analysis consistently associated the archaeological specimens with the Throumbolia morphotype (**Figure 6**). Three additional cultivars, Koutsourelia, Karidolia Chalkidikis and Asprolia Alexandroupolis clustered in the same group but in different sub-groups.

It is essential to assess whether the observed similarity reflects true cultivar lineage continuity or just independent phenotypic convergence because morphological traits can converge independently in olives subjected to similar ecological or selective pressures (Besnard et al., 2018). Several morphological features suggest against simple phenotypic convergence, including the absence of a mucro, moderate elongation, high symmetry and smooth lateral curvature. Collectively, these traits distinguish Throumbolia from elongated cultivars such as Koroneiki, globular morphotypes and mucro-bearing cultivars such as Mastoidis and Koutsourelia (Kostelenos, 2006; Kostelenos, 2011; Barranco et al., 2000; Rallo et al., 2018). The internal cohesiveness of the archaeological stone morphotypes further suggested that the similarity is not superficial or random but reflects a stable morphometric pattern consistent with the modern Throumbolia morphotype.

The convergence of visual observations and quantitative morphometric analyses provides complementary evidence supporting the close morphological affinity between the archaeological endocarps and the modern Throumbolia cultivar. These results are in agreement with previous geometric morphometric studies demonstrating that endocarp shape preserves biologically meaningful information suitable for investigating olive domestication and diversification in archaeological material (Terral et al., 2021). Although this study is based on two-dimensional image analysis, the observed morphometric similarity supports the broader applicability of quantitative morphometric approaches in archaeobotanical research. The agreement between descriptor-based analyses, hierarchical clustering and direct visual comparison might be considered consistent with morphometric continuity between the archaeological material and the modern cultivar. Confirmation of direct lineage relationships will require additional evidence, such as larger archaeological datasets and, where preservation permits, ancient DNA analyses.

### 4.3 Implications for olive domestication and lineage stability

Stone morphology is known to be relatively stable in olive germplasm affected mainly by genetic factors and not the environment (Blazakis et al., 2024), particularly in vegetatively propagated cultivars where clonal reproduction preserves phenotypic identity across generations (Trujillo et al., 2014; Besnard et al., 2018). The observed identity between the ten archaeological specimens and Throumbolia stones suggested that a distinctive morphotype associated with this cultivar was already established at the time represented by the archaeological context. Importantly, the archaeological specimens analyzed in this study do not derive from a single chronological horizon. The carbonized stones from Crete originate from substantially earlier Minoan contexts (Hatzi-Vallianou, 2003), whereas the Tria Platania assemblage corresponds to the Hellenistic period (4th–2nd century BC) (Margaritis and Jones, 2008). The occurrence of a similar morphometric profile across these temporally distant assemblages further supports the long-term stability and continuity of a Throumbolia-like morphotype through time.

Similar results were obtained for grapevine, where both ancient DNA and seed morphometrics have demonstrated that domestication and cultivar-related variation can be preserved over long periods of time (Noraz et al., 2026; Bonhomme et al., 2021). Within a broader archaeobotanical framework, such continuity is consistent with long-term patterns of plant use and cultivation in the Aegean documented from the Neolithic to Classical periods (Megaloudi, 2006).

The low variability observed among the ten archaeological specimens found in two different regions of Greece, Northern Greece and Crete, further supports the interpretation of organized cultivation or management of specific olive germplasm rather than mixed harvesting from diverse sources. Such stability implies continuity of selection and propagation practices that maintained cultivar identity over extended periods.

While morphometric evidence alone cannot resolve questions of genetic ancestry or introgression, the observed morphological identity indicates that at least the phenotypic characteristics of this lineage have remained remarkably stable through time (Besnard et al., 2018).

### 4.4 Methodological considerations and future directions

Despite the results obtained in this report, several methodological issues might be acknowledged. The archaeological dataset was limited to a small number of carbonized endocarps that satisfied the strict preservation criteria required for reliable morphometric analysis. This is particularly relevant for the Cretan assemblage, which was represented by only two archaeological endocarps, precluding conclusions regarding the regional persistence of the observed morphometric profile. An additional issue of this report is that the archaeological specimens span a broad chronological interval, from the Minoan to the Hellenistic period. Consequently, the analyzed endocarps should not be interpreted as representing a single homogeneous population but rather as independent archaeological observations used to evaluate the feasibility of digital morphometric analysis on carbonized olive material. Furthermore, unlike the modern reference database which was generated from images acquired according to the standardized IOC/UPOV protocol in both Position A and Position B, the archaeological material was available only as previously published photographs. Consequently, image acquisition conditions and specimen orientation could not be standardized.

The report should be interpreted primarily as a proof-of-concept approach demonstrating the applicability of the OliveID software to archaeological carbonized olive endocarps. One of the objectives was to evaluate whether reliable morphometric information can be extracted from published images of archaeological material and quantitatively compared with a modern reference database.

Another issue is the use of previously published photographic material rather than the original archaeological specimens. Image resolution and acquisition conditions could not be controlled and might have affected the precision of the extracted morphometric descriptors. The inclusion of the original published photographs as Supplementary Figures allows independent assessment of the preservation quality of the selected specimens and the suitability of the selected endocarps for morphometric analysis. Although strict selection criteria were applied to include only well-preserved archaeological endocarps suitable for reliable contour extraction, the lack of standardized image acquisition should be considered when interpreting the morphometric comparisons.

Additionally, two-dimensional morphometrics cannot fully capture all aspects of endocarp geometry, particularly dorsal–ventral asymmetry and volumetric characteristics (Terral et al., 2004; Bourgeon et al., 2018). Recent advances in three-dimensional (3D) imaging technologies provide complementary morphometric information that may improve the characterization of archaeological olive endocarps and strengthen comparisons with modern cultivars. However, because archaeological material is often available only as published photographs or legacy image collections, robust 2D image analysis remains an important and widely applicable approach for archaeobotanical investigations.

Future studies integrating standardized 3D imaging with the present 2D morphometric framework, together with complementary approaches such as ancient DNA analysis are expected to provide a comprehensive understanding of genetic distances among cultivars and olive domestication history.

This report demonstrates the feasibility of extracting diagnostic geometric information from sufficiently preserved photographic images of archaeological carbonized olive endocarps using the OliveID software and comparing these data quantitatively with a modern morphometric reference database. As a proof-of-concept approach, the observed morphometric similarity with the modern Throumbolia cultivar should be regarded as a preliminary morphometric assessment requiring validation by using larger archaeological datasets and original archaeological specimens acquired under standardized imaging conditions.

## Data Availability

The original contributions presented in the study are included in the article/**Supplementary Material**. Further inquiries can be directed to the corresponding author.
