# Supplementary material for: Application of the OliveID morphometric tool for the identification of archaeobotanical carbonized olive endocarps: evidence for morphological continuity with the modern Throumbolia cultivar
Source: Front Plant Sci. 2026 Aug 4;17:1881563. doi: 10.3389/fpls.2026.1881563 (PMC13483070; doi:10.3389/fpls.2026.1881563)
Supplement: Supplementary file 1 [file DataSheet1.pdf]

Supplementary Figure S3. Schematic overview of the OliveID image analysis workflow.

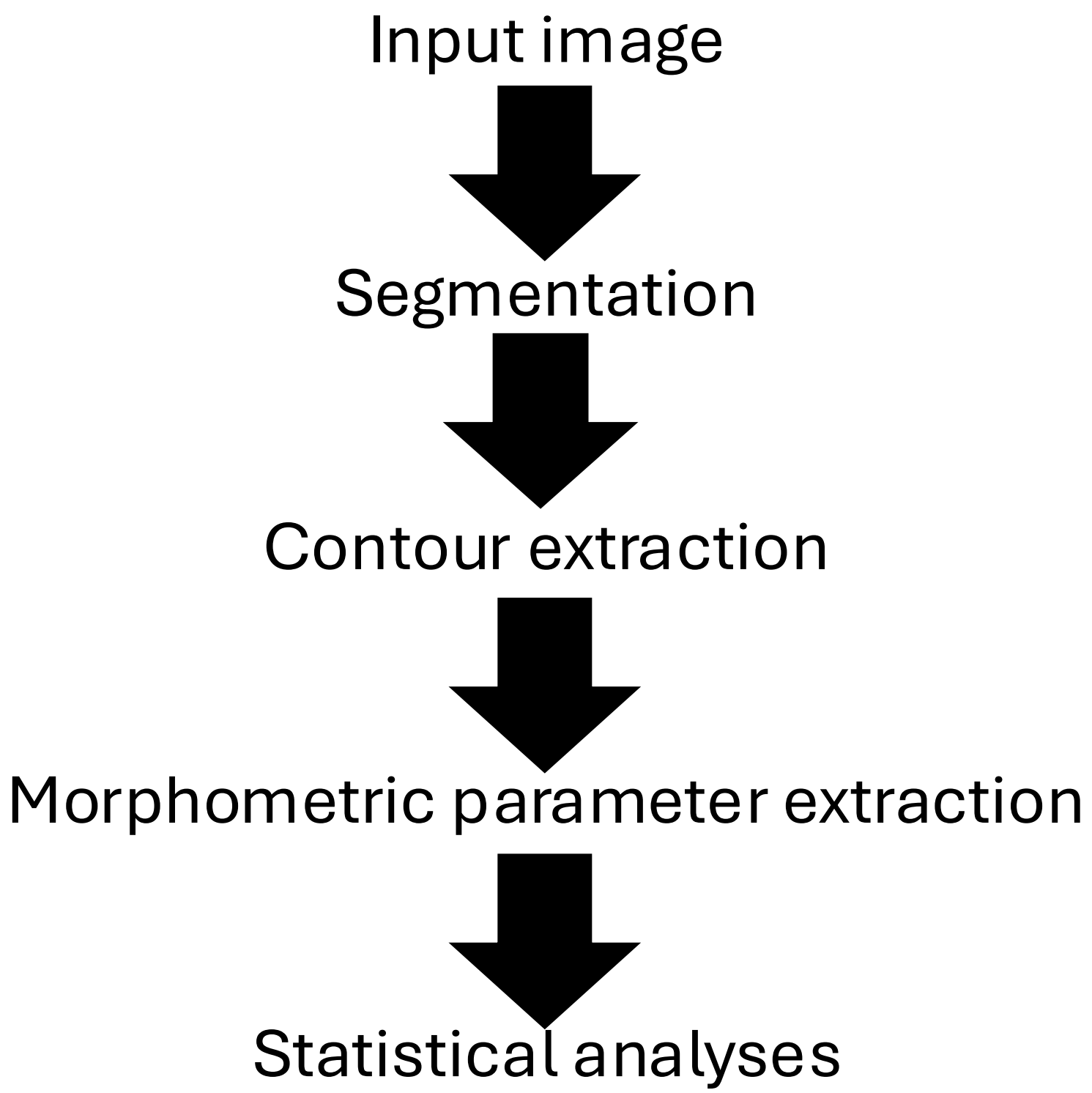

Supplementary Figure S3. Schematic overview of the OliveID image analysis workflow. The workflow consists of (1) image acquisition and preprocessing, (2) image segmentation and background removal, (3) automatic contour extraction, (4) extraction and computation of quantitative morphometric descriptors describing endocarp size, shape, symmetry, curvature and contour geometry, and (5) statistical analyses including hierarchical clustering (UPGMA) and comparative morphometric analyses. A detailed description of the OliveID image-processing workflow is provided in Blazakis et al. (2017).
