# Supplementary material for: Application of the OliveID morphometric tool for the identification of archaeobotanical carbonized olive endocarps: evidence for morphological continuity with the modern Throumbolia cultivar
Source: Front Plant Sci. 2026 Aug 4;17:1881563. doi: 10.3389/fpls.2026.1881563 (PMC13483070; doi:10.3389/fpls.2026.1881563)
Supplement: Supplementary file 2 [file DataSheet2.pdf]

Sample	area-stone	perimeter-st	height-stone	max-tr-diam-	minCTD-stor	vertical-sym	transversal-s	shape-index-	major-axis-st	minor-axis-st	apex-area-st	base-area-st	apex-length-	base-length-	apex-curvatu	base-curvatu	circularity-er	circularity-el	circularity-ratio-stone
CS1	0,62475143	3,0229312	1,26644051	0,63600437	0,30680696	0,47474121	0,48925701	1,99124501	1,24364458	0,62264827	0,025289	0,02448379	0,3436524	0,34434709	0,07277275	0,08313302	0,84646411	0,85846415	0,98602155
CS2	0,51259512	2,73818202	1,20342623	0,63087103	0,30874896	0,55261312	0,47919863	1,90756361	1,18176455	0,61762273	0,021373	0,02007139	0,31592668	0,31177824	0,08610631	0,1014086	0,85641601	0,86841623	0,98618174
CS3	0,61716512	3,00452468	1,02019039	0,6356572	0,31011284	0,53549712	0,42604312	1,60493812	1,00182697	0,6223084	0,018129	0,02405733	0,29096482	0,34133499	0,10151416	0,08460671	0,91591661	0,92791141	0,98706771
CS4	0,56248916	2,86835003	1,02637222	0,63315468	0,31268725	0,60214812	0,46325901	1,62104561	1,00789752	0,61985843	0,015954	0,02199537	0,2729533	0,32637937	0,11535353	0,09253819	0,91179701	0,92379702	0,98701013
CS5	0,46495112	2,60782668	1,18635694	0,62869036	0,3098881	0,59163506	0,40916711	1,88702904	1,16500251	0,61548786	0,016785	0,01809798	0,27997176	0,29605485	0,10964255	0,1124662	0,85536983	0,86736912	0,98616506
CS6	0,50571701	2,71974951	1,41977741	0,63055622	0,30426413	0,47624519	0,49478902	2,25162751	1,39422141	0,61731454	0,02349	0,01982818	0,33120361	0,30988357	0,07834611	0,10265244	0,79502571	0,82125671	0,96954268
CS7	0,52806902	2,77920427	1,22776863	0,63157927	0,30504265	0,48126024	0,44011401	1,94396619	1,20566879	0,61831611	0,021916	0,02060894	0,3199147	0,31592569	0,08397291	0,09876351	0,85425622	0,86625621	0,98614728
CS8	0,54994901	2,83619665	1,18412848	0,63258072	0,30683574	0,50427601	0,46487265	1,87190101	1,16281417	0,61929652	0,023675	0,02150795	0,33250528	0,3227428	0,0777339	0,09463533	0,87202738	0,88402713	0,98642575
CS9	0,56425601	2,87285181	1,03266091	0,63323555	0,30470918	0,46135801	0,49791512	1,63076905	1,01407301	0,61993761	0,018685	0,02212923	0,29539294	0,327371	0,09849345	0,09197843	0,91657325	0,92857312	0,98707694
CS10	0,48675111	2,66826253	1,13040419	0,62968814	0,30330166	0,46664671	0,46559411	1,79518101	1,11005692	0,61646469	0,017249	0,0190375	0,28381511	0,30364214	0,10669315	0,1069159	0,87727261	0,88927212	0,98650582
