# Supplementary material for: Application of the OliveID morphometric tool for the identification of archaeobotanical carbonized olive endocarps: evidence for morphological continuity with the modern Throumbolia cultivar
Source: Front Plant Sci. 2026 Aug 4;17:1881563. doi: 10.3389/fpls.2026.1881563 (PMC13483070; doi:10.3389/fpls.2026.1881563)
Supplement: Supplementary file 3 [file DataSheet3.pdf]

Cultivar	Area Mean	Area SE	Perimeter Me	Perimeter SE	Height Mean	Height SE	Maximum tra	Maximum tra	Minimum dist	Minimum dist	Vertical symr	Vertical symr
arbequina	0,76803756	0,00924423	3,23507706	0,01930189	1,21504418	0,00705019	0,80543523	0,00514381	0,36980838	0,00329746	0,44249913	0,00448794
arbosana	0,73518775	0,00724718	3,25378955	0,01579087	1,2741965	0,00768458	0,73868128	0,00558287	0,32580462	0,00441256	0,43483038	0,00510354
arvanitolia	1,85883797	0,0520838	5,76401478	0,09191506	2,50904221	0,04137115	0,96163956	0,01205316	0,34493425	0,01158035	0,50004372	0,00517488
asprolia-alex	1,07054459	0,01863096	3,80860041	0,03515791	1,42281493	0,01508479	0,99971817	0,01045694	0,43949202	0,00617315	0,49111258	0,00498022
gaidourelia	1,91875572	0,02612787	5,90481294	0,04954162	2,60221491	0,02537938	1,00115007	0,00754827	0,40482109	0,00700199	0,53939114	0,00491807
kalamon	1,47841989	0,0262731	5,06902187	0,0506207	2,17889593	0,02344868	0,89397039	0,00856233	0,33731358	0,00998156	0,4399225	0,00861943
karidolia-cha	1,73325624	0,03093226	5,29561275	0,04962526	2,23551187	0,02257128	1,03314118	0,01184825	0,43114756	0,00848762	0,47495956	0,00557337
karidolia-triz	0,89794704	0,00743575	3,66405254	0,01639746	1,49251784	0,00762965	0,82375465	0,00383249	0,35140536	0,00424821	0,48709096	0,00369067
koroneiki	0,61945241	0,0056715	3,09126097	0,01717638	1,29449587	0,0091327	0,64614787	0,00289113	0,27778411	0,0031815	0,47885166	0,00392308
kothreiki	1,09364996	0,01090497	4,01766539	0,02406796	1,60660441	0,01245083	0,90171571	0,00494764	0,39500308	0,00406983	0,47504983	0,00693328
koutsourelia	0,75194127	0,01236482	3,45904588	0,03296795	1,46695001	0,01617081	0,73935502	0,00604721	0,30798402	0,00483541	0,51500184	0,00413689
lefkolia-serrc	1,53572142	0,01824737	4,7880676	0,03339461	1,9294889	0,0180467	1,04450994	0,00928661	0,44306825	0,00828099	0,51253605	0,00604721
lianomanako	0,77859992	0,00829801	3,33500409	0,02053346	1,30882507	0,00943553	0,75347854	0,00390697	0,34348296	0,00262697	0,44491656	0,0062015
mastoidis	0,78577275	0,01061965	3,72271355	0,02917288	1,63823188	0,01474136	0,65470017	0,00464984	0,2579118	0,00651064	0,51177562	0,00567963
mastoidis-n-	0,82573785	0,00912527	3,64918455	0,03017184	1,546697	0,01684751	0,77297988	0,00324416	0,31863661	0,00398523	0,548934	0,00343231
mavrolia-ser	1,3250571	0,02196872	4,28362581	0,03631688	1,63723029	0,0157297	1,04588248	0,00909355	0,47914778	0,00620079	0,50957026	0,00528716
megaron	1,00485597	0,0134486	4,08417721	0,03286217	1,74686991	0,01638386	0,82078169	0,00550215	0,34204559	0,00462467	0,55356987	0,00362761
mothonia-eg	0,92989295	0,00938209	3,80955952	0,02373844	1,5848524	0,01248211	0,77898598	0,0037408	0,32470706	0,00391546	0,45914311	0,00446524
ntopia-atsihc	0,84190449	0,01130917	3,66097281	0,02692306	1,56160586	0,01308457	0,73845828	0,00496084	0,31742662	0,00452128	0,48429055	0,00371102
plexidenia	0,86351502	0,00906389	3,5913577	0,02098876	1,48322137	0,01059497	0,77981233	0,00398283	0,33020491	0,00386117	0,53971665	0,00327942
rahati	0,81449113	0,01356577	3,67582863	0,04016454	1,57876796	0,02126954	0,73776515	0,00684717	0,27623556	0,00740438	0,49096713	0,00575433
smertolia	0,97940112	0,01430412	3,69719103	0,0247775	1,43306892	0,01018712	0,8670101	0,00888628	0,40697695	0,0049937	0,54170072	0,00687025
thiaki	0,70831823	0,0084855	3,29229577	0,0222715	1,36584412	0,01034929	0,70562827	0,00426947	0,2970653	0,00333919	0,44427249	0,00388647
throubolia	1,07493477	0,02156886	3,97369846	0,03722193	1,62414538	0,01529759	0,88676181	0,00985369	0,38645538	0,00554269	0,51251644	0,00367811
tragolia	0,62701588	0,0055353	3,02488441	0,01400466	1,21851056	0,00749885	0,68847199	0,00402059	0,2903817	0,00370138	0,44641289	0,00431649

Cultivar	Transversal s	Transversal s	Shape index I	Shape index S	Major axis of	Major axis of	Minor axis of	Minor axis of	Area of the ap	Area of the ap	Area of the ba	Area of the ba	Length of the
arbequina	0,45892067	0,00256201	1,51125306	0,00693403	1,20895014	0,0072866	0,81026207	0,00546247	0,0206393	0,00026143	0,01281745	0,00030268	0,35013141
arbosana	0,44001579	0,0042359	1,7368433	0,01708014	1,26886276	0,00734065	0,74364658	0,00583924	0,02129231	0,00025794	0,01582483	0,00039806	0,33475247
arvanitolia	0,35638534	0,01061328	2,60254786	0,02270586	2,48766326	0,04256737	0,96469442	0,01235118	0,06570829	0,00177606	0,08779646	0,00272997	0,4540992
asprolia-alex	0,43945378	0,00384797	1,43108178	0,01434054	1,37717354	0,01378716	0,99038062	0,01037844	0,02572	0,000519	0,02501243	0,00080016	0,41109472
gaidourelia	0,40374892	0,00600056	2,60849489	0,02589699	2,51733934	0,02362632	0,98585817	0,00746248	0,0759964	0,00123105	0,08666666	0,00146816	0,50369634
kalamon	0,37518827	0,00991647	2,4447264	0,02235789	2,16345023	0,02435742	0,89581521	0,00845372	0,06200906	0,00127761	0,06148188	0,00134022	0,40409475
karidolia-cha	0,41487525	0,00505103	2,1792543	0,02152982	2,16646441	0,02151762	1,02736288	0,01131957	0,05766893	0,00116765	0,06230934	0,00146964	0,45892856
karidolia-triz	0,42630482	0,00453616	1,81433738	0,00982468	1,42780585	0,00715353	0,81290357	0,00393915	0,02747253	0,00028698	0,0271167	0,00040543	0,3182191
koroneiki	0,42945783	0,00422861	2,00740299	0,01562875	1,24646202	0,00883016	0,63962698	0,00293118	0,0212589	0,00028448	0,02134309	0,0003462	0,27507023
kothreiki	0,43806356	0,00384902	1,78610783	0,0153043	1,56184749	0,01149696	0,90106585	0,00490295	0,03193201	0,0005211	0,02928021	0,00062406	0,38421743
koutsourelia	0,41597914	0,00521813	1,98672673	0,01694268	1,35883075	0,01425338	0,71989394	0,0058212	0,02615286	0,00049965	0,02791942	0,00048525	0,30866618
lefkolia-serrc	0,42161086	0,00536997	1,86328376	0,02360542	1,89949475	0,01733235	1,03940095	0,00913285	0,04760667	0,00079686	0,04669482	0,00125503	0,49521914
lianomanako	0,4559516	0,00262432	1,73933976	0,01189835	1,31017974	0,00989456	0,76020394	0,00390794	0,02371319	0,00036315	0,01738648	0,00036508	0,35457127
mastoidis	0,39396905	0,00955862	2,50749433	0,02507983	1,56519325	0,01464877	0,65176457	0,0047758	0,02851963	0,00047716	0,0347899	0,00062479	0,30088478
mastoidis-n-	0,41239218	0,00496858	2,0005274	0,01993974	1,43886002	0,01410605	0,7506256	0,00307528	0,02929069	0,0005757	0,03033446	0,00051593	0,33728257
mavrolia-ser	0,45711667	0,00329124	1,56918679	0,01229506	1,6185892	0,01530165	1,04011859	0,00909086	0,03260437	0,00068845	0,03101786	0,00084859	0,47940335
megaron	0,41648286	0,00467036	2,12946976	0,0164216	1,64150587	0,01405559	0,79581187	0,0052543	0,03063805	0,00050679	0,04317207	0,00066619	0,37783086
mothonia-eg	0,4168339	0,00461418	2,03762164	0,01613776	1,54618937	0,01205843	0,77830558	0,00373288	0,03081954	0,00042372	0,02928172	0,00065905	0,32391665
ntopia-atsihc	0,42901156	0,00493212	2,1169016	0,01321126	1,4898599	0,01165543	0,7246265	0,00469916	0,02943685	0,00039504	0,03411441	0,00060295	0,33790971
plexidenia	0,42324808	0,00424553	1,90242969	0,01021711	1,4313816	0,00923617	0,77220785	0,00380767	0,02642676	0,00038147	0,02931589	0,00040933	0,35921053
rahati	0,37357257	0,00856778	2,14457461	0,02843257	1,47342537	0,01878314	0,72531357	0,00634718	0,02943617	0,00065338	0,03096265	0,00061699	0,29841546
smertolia	0,46919655	0,00262262	1,66115898	0,01780706	1,43151963	0,01015212	0,87196402	0,00891582	0,02383397	0,00037387	0,02512873	0,00060756	0,43375078
thiaki	0,42134816	0,00430336	1,93710856	0,0111483	1,31008519	0,00969072	0,6976655	0,00420796	0,02295856	0,00025859	0,0233621	0,00038041	0,28085236
throubolia	0,43568423	0,0037889	1,84186468	0,01393502	1,56228609	0,014729	0,87492842	0,01001212	0,03124785	0,00063884	0,03392502	0,00073456	0,38330853
tragolia	0,42104308	0,00417204	1,7758306	0,01427592	1,17615408	0,00669447	0,68674611	0,00401988	0,01938231	0,00021728	0,01758858	0,00031111	0,28228975

Cultivar	Length of the	Length of the	Length of the	Average curv	Average curv	Average curv	Average curv	Average circ	Average circ	Average circ	Average circ	Circularity ra	Circularity ratio SE
arbequina	0,00277608	0,39528938	0,00249639	0,01697008	0,0002435	0,01138027	0,00025853	0,91826471	0,00119219	0,9414714	0,00120435	0,97537044	0,00060543
arbosana	0,00245729	0,38775311	0,00234218	0,01885	0,00024616	0,01398647	0,00026016	0,87072458	0,00330538	0,89775869	0,00326797	0,96985918	0,0008235
arvanitolia	0,006452	0,55268241	0,01004305	0,01461704	0,00021126	0,01573494	0,00021344	0,69335919	0,00425489	0,73444502	0,00406839	0,94384851	0,00161145
asprolia-alex	0,0042935	0,43952014	0,00411399	0,01492382	0,00031616	0,01345498	0,00038197	0,92029372	0,00279379	0,95679775	0,00229213	0,96175716	0,00133069
gaidourelia	0,00415173	0,51517139	0,0054088	0,01500761	0,00027821	0,01569878	0,00027059	0,69156238	0,00461982	0,73607372	0,00449447	0,93934646	0,00181111
kalamon	0,00544014	0,51050027	0,00547487	0,01891081	0,00043988	0,01702344	0,00032397	0,71959989	0,00417737	0,76100022	0,00407043	0,9455272	0,00191057
karidolia-cha	0,00584584	0,54920851	0,00626849	0,01496219	0,00025709	0,01328726	0,00026391	0,77107013	0,00378795	0,8172454	0,00418551	0,94380383	0,00187064
karidolia-triz	0,00283658	0,41152575	0,00309111	0,02026361	0,00036464	0,01807961	0,00029545	0,8396955	0,0027683	0,88992163	0,00185035	0,94335962	0,00157634
koroneiki	0,00191036	0,33109849	0,00215397	0,02312175	0,00042021	0,02135302	0,00033938	0,81442094	0,00325122	0,85084345	0,00307131	0,95709773	0,00108906
kothreiki	0,00446812	0,45601665	0,00338523	0,01886361	0,00048055	0,01469366	0,0003742	0,85076813	0,00339564	0,89416886	0,00277562	0,95125813	0,00159074
koutsourelia	0,00303908	0,28068509	0,00288925	0,02047468	0,00033205	0,02312256	0,0002765	0,78704439	0,00406581	0,863128	0,00321654	0,91146083	0,00214253
lefkolia-serrc	0,00352373	0,52344832	0,00463323	0,01520089	0,00029447	0,01421356	0,00029025	0,84121941	0,00491333	0,87240305	0,00466984	0,96405874	0,00128751
lianomanako	0,00195507	0,39790556	0,00257203	0,01881148	0,0002266	0,01379018	0,00024322	0,87789654	0,00227297	0,89661873	0,00232493	0,97915091	0,00069396
mastoidis	0,00413574	0,31370662	0,00416271	0,02314563	0,00033809	0,02288563	0,00034095	0,71220748	0,00490974	0,76324117	0,00432241	0,93288428	0,00230659
mastoidis-n-	0,00244498	0,2812365	0,00312758	0,02134177	0,00045336	0,02299688	0,00047759	0,78179422	0,00519168	0,85789955	0,00362031	0,91029136	0,00260286
mavrolia-ser	0,00433056	0,50099712	0,00470362	0,0135343	0,00028129	0,01251634	0,00032329	0,90206496	0,00263526	0,92882196	0,00234695	0,97115204	0,00113121
megaron	0,00514015	0,34118651	0,00278492	0,02022625	0,00052807	0,02489298	0,00053358	0,75635879	0,00438392	0,82851731	0,00284492	0,91226184	0,00271925
mothonia-eg	0,00235262	0,42935222	0,00248124	0,02007664	0,00026619	0,01655358	0,00024819	0,80479456	0,00327993	0,84347466	0,00308218	0,95401386	0,00099383
ntopia-atsihc	0,00272238	0,34675474	0,00241428	0,02032563	0,00029635	0,02142031	0,00027022	0,78725204	0,00314417	0,82955517	0,00249912	0,94873898	0,00144852
plexidenia	0,0024848	0,37067969	0,00245146	0,01921594	0,00037676	0,02177736	0,00032569	0,83975969	0,0024983	0,87061433	0,00181124	0,96438627	0,00120704
rahati	0,00333442	0,29888063	0,00473782	0,02331776	0,00038744	0,02359326	0,00035191	0,75810593	0,00653469	0,83428961	0,00515857	0,90810948	0,00309042
smertolia	0,00336557	0,4178844	0,00399533	0,01685272	0,00034967	0,01708142	0,0003718	0,89773318	0,00338395	0,91157208	0,00330674	0,98479415	0,00047093
thiaki	0,00239782	0,36095269	0,00273701	0,022532	0,00036479	0,01912251	0,00022864	0,81892981	0,00263594	0,86556009	0,00213602	0,94597805	0,00124994
throubolia	0,0050357	0,43567984	0,00505438	0,01853235	0,00034283	0,01852179	0,00032439	0,84546681	0,00331882	0,88228456	0,00263243	0,95799814	0,00133213
tragolia	0,00260982	0,33982396	0,00232669	0,02243017	0,00031309	0,01835326	0,00028136	0,86019154	0,00324587	0,89793405	0,00264816	0,95777024	0,00129247
