# Supplementary material for: Application of the OliveID morphometric tool for the identification of archaeobotanical carbonized olive endocarps: evidence for morphological continuity with the modern Throumbolia cultivar
Source: Front Plant Sci. 2026 Aug 4;17:1881563. doi: 10.3389/fpls.2026.1881563 (PMC13483070; doi:10.3389/fpls.2026.1881563)
Supplement: Supplementary file 4 [file Supplementaryfile1.pdf]

No.	Descriptor	Abbreviation	Description	Morphological information
1	Area	area-stone	Projected area of the endocarp	Overall size
2	Perimeter	perimeter-stone	Total contour length	Overall size and contour complexity
3	Height	height-stone	Maximum longitudinal length	Endocarp length
4	Maximum transversal diameter	max-tr-diam-stone	Maximum width perpendicular to the longitudinal axis	Endocarp width
5	Minimum contour–transversal diameter distance (minCTD)	minCTD-stone	Minimum distance between the maximum transversal diameter and the contour	Cross-sectional contour geometry
6	Vertical symmetry	vertical-symmetry-stone	Symmetry with respect to the longitudinal axis	Bilateral symmetry
7	Transversal symmetry	transversal-symmetry-stone	Symmetry with respect to the transversal axis	Cross-sectional symmetry
8	Shape index	shape-index-stone	Height / maximum transversal diameter	Degree of elongation
9	Major ellipse axis	major-axis-stone	Length of the major axis of the fitted ellipse	Overall elongation
10	Minor ellipse axis	minor-axis-stone	Length of the minor axis of the fitted ellipse	Overall width
11	Apex area	apex-area-stone	Area enclosed by the apex region	Apex morphology
12	Base area	base-area-stone	Area enclosed by the base region	Base morphology
13	Apex length	apex-length-stone	Length of the apex region	Apex geometry
14	Base length	base-length-stone	Length of the base region	Base geometry
15	Apex curvature	apex-curvature-stone	Mean curvature of the apex	Apex shape
16	Base curvature	base-curvature-stone	Mean curvature of the base	Base shape
17	Endocarp circularity	circularity-endocarp-stone	Circularity of the endocarp contour	Overall compactness
18	Ellipse circularity	circularity-ellipse-stone	Circularity of the fitted ellipse	Ellipticity
19	Circularity ratio	circularity-ratio-stone	Ratio between endocarp and fitted ellipse circularity	Deviation of the endocarp from an ideal ellipse
